# Assessment of lipid composition and eicosapentaenoic acid/docosahexaenoic acid bioavailability in fish oil obtained through different enrichment methods

**DOI:** 10.3389/fnut.2023.1136490

**Published:** 2023-03-14

**Authors:** Rongzhen Song, Wen Li, Shanggui Deng, Yueliang Zhao, Ningping Tao

**Affiliations:** ^1^College of Food Science and Technology, Shanghai Ocean University, Shanghai, China; ^2^College of Food and Pharmacy, Zhejiang Ocean University, Zhoushan, Zhejiang, China; ^3^Shanghai Engineering Research Center of Aquatic-Product Processing and Preservation, Shanghai, China; ^4^National Experimental Teaching Demonstration Center for Food Science and Engineering, Shanghai Ocean University, Shanghai, China

**Keywords:** eicosapentaenoic acid (EPA), docosahexaenoic acid (DHA), lipids composition, Caco-2 cell, bioavailability

## Abstract

In this study, we analyzed the eicosapentaenoic acid/docosahexaenoic acid (EPA/DHA) lipid composition of fish oil obtained through enzymatic treatment, fractional distillation and silica gel column purification, and further assessed EPA/DHA bioavailability. Lipid subclass composition information was obtained through ultra-performance liquid chromatography-electrospray ionization tandem mass spectrometry (UPLC-ESI-MS/MS), and bioavailability tests were performed using the Caco-2 cell monolayer model. Results showed that enzymatic treatment improved the incorporation of EPA/DHA as diacylglycerol (DG) while silica gel column chromatography enriched the content of EPA/DHA as phosphatidylglycerol (PG) (12.58%) and phosphatidylethanolamine (PE) (4.99%). Furthermore, increasing the purity of EPA/DHA could improve its bioavailability and after 24 incubation, binding forms of triglyceride (TG) was superior to ethyl ester (EE) (*p* < 0.05) at the same purity level. Those findings are helpful to provide research basis for exploring the bioactivity of fish oil.

## Introduction

Fish oils are rich in ω-3 polyunsaturated fatty acids (PUFAs) such as eicosapentaenoic acid (EPA, 20:5) and docosahexaenoic acid (DHA, 22:6), and are known to be essential for human health. EPA/DHA has beneficial effects on cardiovascular disease and whereby it can prevent the oxidation of ApoB-containing particles by facilitating the clearance of low-density lipoprotein (LDL) (1). ω-3 17, 18-epoxide of EPA is involved in anti-proliferative and proapoptotic signaling cascades in tumor cells (2). Current studies also suggest that supplementation with fish oil rich in EPA/DHA could protect against bone loss and regulate bone metabolism (3). EPA has also been reported to be beneficial for treating anxiety and depressive disorders by increasing neuronal membrane fluidity (4). Furthermore, previous studies have reported that regular consumption of fish oils rich in EPA/DHA is associated with reduced risk of Alzheimer’s and Parkinson’s disease (5, 6).

The EPA/DHA content in natural fish oil is around 30%, which is deemed as having low nutritional value and minor economic benefits. In recent years, there have been many reports focus on enriching EPA/DHA of fish oil to improve the functional quality and enhance the market competitiveness. Typically, enzyme-based processes are always tested under mild conditions as a means to improve the incorporation of EPA/DHA as triglycerides (TGs). Gao et al. (7) improved the EPA/DHA content from 14.95 to 41.84% in codfish TGs using *Streptomyces violascens* OUC-Lipase 6 which selectively hydrolyzes fatty acids on the glyceride backbone to increase the contents of DHA and EPA. Cao et al. (6) obtained fish oil with 48.35% EPA/DHA by means of lipase catalysis. Moreover, fractional distillation is especially suitable for the separation of substances with high boiling points and easily to be oxidized, which are usually applied to incorporate EPA/DHA as ethyl esters (EEs). Zhang et al. (8) used response surface methodology to optimize the purification processes and successfully improved the EPA/DHA content as EEs up to 78.12%. In another study, Zhou et al. (9) obtained 96% DHA incorporated as EEs through fractional distillation. Silica gel column chromatography, a traditional separation method, was also used to improve the EPA/DHA purity and takes advantage of the lipid molecules polarity to enable separation. Compared to enzymatic-based methods, silica gel column purification was superior as this approach protected the natural structure as TGs from destruction and was also simple to operate.

Bioavailability of EPA/DHA as TGs as well as EEs has been a controversial issue, and many studies have focused on this (10). In general, EPA/DHA as EEs are believed to be less efficient because of the undesirable hydrolysis rate of carboxyl ester lipase (11–13). Generally, human trials to assess bioavailability are regarded as “the gold standard”. However, Neubronner et al. (13) contended that plasma levels of EPA/DHA do not adequately reflect their incorporation into tissues. Furthermore, they also argued that the percentage of EPA/DHA in red blood cell membranes is the endpoint to assess bioavailability, as it can indicate the integration of EPA/DHA into tissues. But there were many limitations for human trials, such as high-cost, time-consuming, and have ethical controversy. In recent years, *in vitro* digestion combined with the Caco-2 cell model has been commonly applied to investigate the bioavailability of functional substances in food (14–17). This approach also offered the advantages of lower costs, ease of operation, and larger sample size.

In this study, we prepared fish oil with high purity of EPA/DHA as both TGs and EEs through enzymatic reactions, fractional distillation, and silica gel column separation, followed by analyzing the EPA/DHA lipid subclasses composition through ultra-high-performance liquid chromatography-tandem mass spectrometry (UPLC-MS/MS). To investigate the bioactive properties of EPA/DHA more thoroughly and provide new theoretical foundations that may be applied to the food industry, we further analyzed the effect of differences in purity on EPA/DHA bioavailability using the Caco-2 cell model. The results from this work could boost the enzymatic inter-esterification technology to significantly enhance EPA/DHA purity and bioavailability as TG products, overcome the challenges of poor digestion and absorption of EPA/DHA as EEs, and further demonstrate industrial applications of the improved products for the aquatic oil processing industry. Column chromatography was explored as a strategy to purify EPA/DHA with high bioavailability and we discuss if this approach is also a promising process for industrialization.

## Materials and methods

### Materials

**FIGURE 1:**
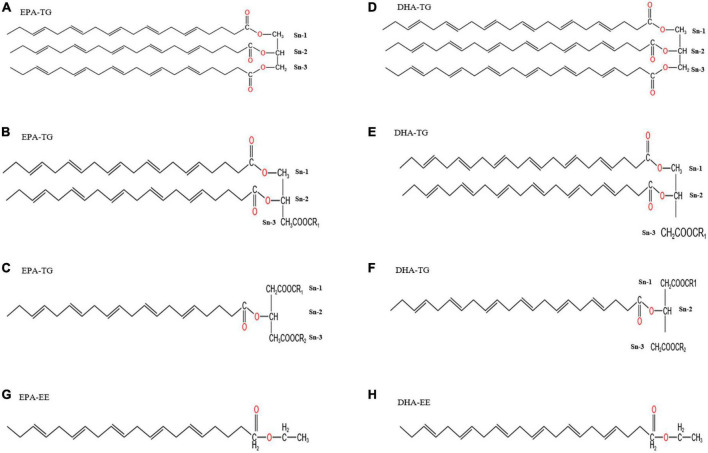
Chemical structure diagrams of EPA/DHA as TGs and EEs. Panels **(A–F)** show the chemical structures of EPA/DHA as TGs, indicating that it is possible for EPA/DHA to locate at *sn*-1, *sn*-2, and *sn*-3 in TGs. EPA/DHA in fish oil is more likely to locate at *sn*-2. Every TG could have one, two or three EPA/DHA. Panels **(G,H)** show the chemical structures of EPA/DHA as EEs.

Natural anchovy oil TG30 (level 1 of refined fish oil according to the SC/T 3502-2016) with about 30% purity of EPA/DHA; ethyl esterified fish oil EE30 (obtained from TG30 by esterification at an esterification rate of > 80%) with about 30% purity of EPA/DHA; rTG50 and rTG70 refer to the TG30 re-esterified with 50 and 70% EPA/DHA purity, respectively; EE50 and EE70 are derived from EE30 subjected to fractional distillation resulting in 50 and 70% purities of EPA/DHA as EEs, respectively. All products were all kindly provided by Sinomega Biotech Engineering Co. Ltd. (Zhejiang, China). We improved the purity of EPA/DHA to 54.27% through silica gel (70-230 mesh, Merck, Darmstadt, Germany) column separation from TG30 named TG50. More importantly, this enrichment method could protect fish oil natural molecular structure from destroying compared with enzymatic treatment. Briefly, the TG50 fraction was eluted using n-hexane/ethyl acetate, 1:20 (v/v). The eluent was collected and the organic reagent was removed by rotary evaporation at 40°C at 90 rpm (18). The rest of the total lipids were collected and stored at −40°C for later use. The purity of EPA/DHA and fatty acid composition for each fish oil sample is presented in Table 1. More importantly, EPA/DHA purity was defined as the fractions of EPA/DHA concentration versus the total fatty acid (TFA) concentration. Saturated fatty acid (SFA), monounsaturated fatty acid (MUFA), and PUFA relative content were described as the fractions of SFA, MUFA, and PUFA concentrations versus the TFA concentration, respectively. Chemical structures of EPA/DHA as TG and EE are shown in Figure 1.

**TABLE 1:** EPA/DHA purity and fatty acid composition of each fish oil sample.

Samples	EPA/DHA purity (%)	SFA R. C. (%)	MUFA R. C. (%)	PUFA R. C. (%)
TG30	34.08 ± 0.91^c^	30.96 ± 0.26 ^a^	24.50 ± 0.42 ^a^	44.54 ± 0.55 ^f^
TG50	54.27 ± 0.24^b^	16.67 ± 0.09 ^c^	13.55 ± 0.15 ^d^	69.78 ± 0.12 ^b^
rTG50	53.36 ± 1.07^b^	22.23 ± 1.63 ^b^	13.52 ± 0.11 ^d^	64.26 ± 1.68 ^c^
rTG70	74.08 ± 0.53^a^	5.57 ± 0.14 ^d^	11.09 ± 0.24 ^e^	83.34 ± 0.21^a^
EE30	34.31 ± 0.47^c^	30.11 ± 1.04 ^a^	23.29 ± 0.83 ^b^	46.60 ± 0.66 ^e^
EE50	54.09 ± 0.16^b^	16.69 ± 0.11 ^c^	21.20 ± 0.39 ^c^	62.10 ± 0.44 ^d^
EE70	73.88 ± 1.37^a^	3.67 ± 0.22 ^e^	13.59 ± 0.35 ^d^	82.74 ± 0.56 ^a^

Values are mean ± SD, *n* = 3. Values with different superscripts in same row differ significantly (*p*<0.05). Three EPA/DHA purities levels were set at 30, 50, and 70%, and performed significant differences. R. C. means relative content.

Organic solvents (chromatographic grade); Tween 80 was purchased from Shanghai Macklin Biochemical Co., Ltd. C19:0 and the mixed standard of 37 fatty acid methyl esters were all purchased from ANPEL Laboratory Technologies (Shanghai) Inc; minimum essential medium (MEM), fetal bovine serum (FBS), glutamate, non-essential amino acids, sodium pyruvate were all obtained from Gibco Life Technologies (Grand Island, NY, USA).

### Fatty acid analysis

Fatty acid composition in each fish oil sample was determined by gas chromatography (GC) and carried out according to the method described by Zhang et al. (18).

### UPLC-MS/MS to analyze lipid classes

Reverse-phase chromatography was selected for separation with a charged surface hybrid (CSH) C18 column (1.7 μm, 2.1 mm × 100 mm, Waters). Mobile phase A was acetonitrile-water (6:4, v/v) with 0.1% formic acid and 0.1 mM ammonium formate, while the mobile phase B was acetonitrile-isopropanol (1:9, v/v) with 0.1% formic acid and 0.1 mM ammonium formate. The initial mobile phase was 70% solvent A and 30% solvent B at a flow rate of 300 uL/min. This was held for 2 min, and then linearly increased to 100% solvent B in 23 min, followed by equilibrating at 5% solvent B for 10 min.

Mass spectra were acquired through The Q Exactive™ Plus Hybrid Quadrupole-Orbitrap™ Mass Spectrometer (Thermo Fisher Scientific, USA) in both positive and negative mode. The mass spectrometer was operated as follows: ion spray voltage was set at 3,000 V, source temperature 300°C, capillary temperature 350°C, and scan mode ranged from m/z 200 to 1,800. “LipidSearch” software (Thermo Scientific, USA) was used for the identification of lipid classes based on MS/MS math.

### Preparation of fish oil emulsion

An aqueous phase was prepared by dispersing 0.2% (w/w) of Tween 80 into distilled water. The oil phase (fish oil of TG30, TG50, rTG50, rTG70 and EE30, EE50, EE70) (25%, w/w) and the aqueous phase (75%, w/w) were then mixed. The mixture was homogenized for 3 min at 8000 rpm (RV10, IKA, Staufen, Germany) under room temperature, and then subjected to ultrasonic emulsification at 300 W (JT88-IIN, Ningbo Scientz Biotechnology CO., LTD.) for 5 min, after which, an emulsion was formed (19, 20).

### Preparation of *in vitro* digested samples

To provide an environment that was consistent and similar to *in vivo* animal studies and human experiments, fish oil emulsions was subjected to a simulated gastrointestinal tract (GIT), which included the mouth, stomach, and small intestine three stages. Detailed methods were conducted according to the methods of Lin et al. (21) and Sarkar et al. (22) with some modifications. The initial fish oil emulsion mixed with simulated saliva fluid (SSF) at the ratio of 1:1, shaken at 100 rpm at 37°C for 2 min. At the stomach stage, the digestive fluid from mouth stage mixed with simulated gastric fluid (SGF) at the ratio of 1:1, the mixture was shaken at 100 rpm at 37°C for 2 h. At the small intestine stage, the digestive fluid from the stomach stage mixed with simulated intestinal fluid (SIF) also at the ratio of 1:1, the mixture was shaken at 100 rpm at 37°C for 2 h. Preparations of SSF, SGF, and SIF were according to Peaña - Vázquez et al. (23). The digesta were collected after GIT simulation and centrifuged (H1850R, Cence, Hunan, China) at 10,614 g for 55 min at 4°C to obtain the fish oil-enriched mixed micelle phase. Then the mixed micelles were injected through a 0.45 μm syringe filter and stored at −40°C until used for the Caco-2 cells monolayer transport assay. Assessment of EPA/DHA bioaccessibility is not discussed in this article.

### Bioavailability assay

#### Caco-2 cell culture

Caco-2 human colon carcinoma epithelial cells (passages 30-40) were kindly provided by the Stem Cell Bank, Chinese Academy of Sciences, China. The cells were cultivated in 25 cm^2^ flasks with MEM supplemented with 20% FBS, 1% glutamate, 1% non-essential amino acids, and 1% sodium pyruvate solution, and incubated at 37°C with 5% CO_2_ (Forma 310. Thermo Fisher Inc.) (17). The culture medium was changed every 48 h and the cells were sub-cultured when they attained 80-90% confluence, treated with 1 mL 0.25% trypsin-EDTA for about 4 min, and then seeded at a 1:3 dilution into new 25 cm^2^ flasks (16).

#### Cell viability assay

The fish oil mixed micelles were collected and diluted with complete medium to obtain diluents with different concentrations (1%, 10%, 20%, v/v). Cell viability was determined with the Cell-Counting Kit-8 (CCK-8, Dojindo Laboratories, Shanghai, China), which is a sensitive, convenient, and non-toxic method (14). More importantly, Before the addition of CCK-8, we washed the cells for three times by phosphate buffer saline (PBS).

Cells at a density of 1.0 × 10^5^ cells/cm^2^ were seeded into 96-well plates and cultured for 24 h at 37°C. After changing the medium, 10 μL mixed micelles at various concentrations were added and were incubated for 24 h at 37°C. Finally, 10 μL of CCK-8 solution was added and the cells were incubated for an additional 4 h at 37°C. Cell viability was calculated according to the absorbance measured at 450 nm.

#### Cell transport studies

Caco-2 cells were seeded in each transwell (12 wells, 0.4 μm, Labselect, Anhui, China) at a density of 1.0 × 10^5^ cells/cm^2^ and grown for 21 days (24). The trans-epithelial electrical resistance (TEER) was measured using a Millicell ERS-2 (World Precision Instruments, Sarasota, Fl., USA) on day 0, 3, 6, 9, 12, 15, 18, 21 until the TEER values reached above 500 Ω^⋅^cm^2^ (16, 24).

The transport test was performed from the apical chamber to the basolateral chamber. Micelles (0.5 mL of 20%, v/v) were added in the apical part and 1.5 mL of fresh medium (containing 20% FBS, 1% glutamate, 1% non-essential amino acids, 1% sodium pyruvate solution, and 77% MEM, v/v) was added to the basolateral part. After incubating for 0, 6, and 24 h at 37°C, the apical and basolateral solutions were collected to determine the content of EPA/DHA. Meanwhile, the TEER was measured at 0, 3, 6, 12, and 24 h to assess the influence of the micelles on the integrity of the cell monolayer. The results of EPA/DHA bioavailability were expressed as the percentage of the concentration of EPA/DHA in the basolateral medium to the concentration of EPA/DHA in the mixed micelles fractions (25). Moreover, cellular uptake of EPA/DHA is another important index for bioavailability assessment and was expressed as the percentage of EPA/DHA content in the cells relative to the content in the micelles (26). To measure the levels of EPA/DHA in the cellular function after transport at 0, 6, and 24 h and removal of the media, the cell monolayer was washed 3 times with ice-cold PBS to remove free samples. Then cells were collected and treated with PBS containing 15% ethanol to dissociate cell monolayers (27). The organic reagent within the mixture was removed by rotary evaporation at 40°C at 90 rpm. The EPA/DHA was calculated as described in 2.2.

### Statistical analysis

All results of these experiments are presented as mean ± standard deviation (SD). Data were evaluated by one-way analysis of variance (SPSS software, version 16.0, IBM Corporation, Chicago, IL, USA). Multiple comparisons were assessed by Duncan’s test to determine if differences were significant (*p*<0.05).

## Results and discussion

### Composition of lipids class

**FIGURE 2:**
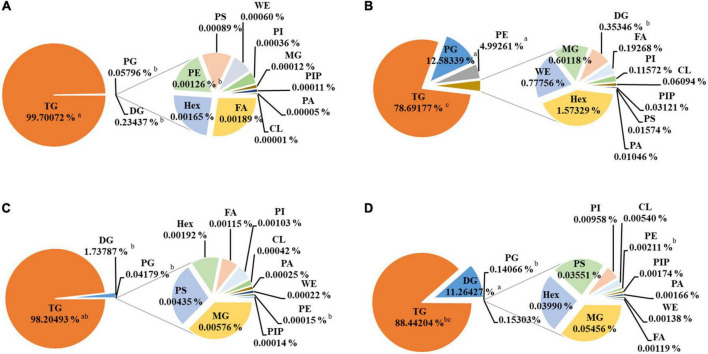
Composition of EPA/DHA lipids subclasses composition. **(A)** TG30, **(B)** TG50, **(C)** rTG50, **(D)** rTG70. Data are represented as mean ± SD (*n* = 3). Different letters represent significant differences (*p* <0.05). Triacylglycerol, TG; diacylglycerol, DG, monoglyceride, MG; phosphatidylglycerol, PG; phosphatidylglycerol, Hex; phosphatidylserine, PS; phosphatidylinositol, PI; cardiolipin, CL; phosphatidylethanolamine, PE; phosphatidylinositol (4) phosphate, PIP; phosphatidic acid, PA; wax exters, WE; fatty acid, FA.

**FIGURE 3:**
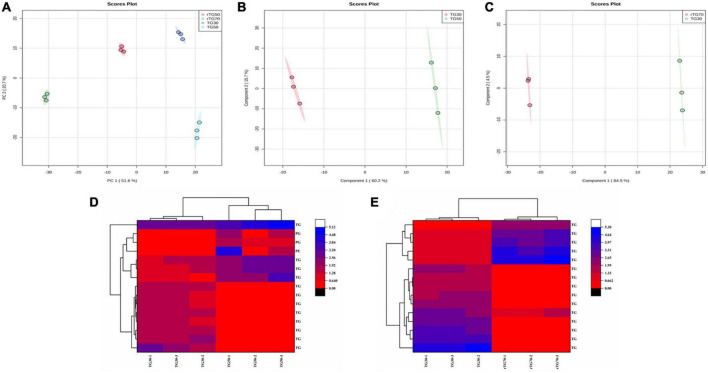
The statistical analysis of EPA/DHA lipid molecules. **(A)** PCA score plots (TG30, TG50, rTG50, and rTG70); **(B)** PLS-DA score plots (TG30 and TG50); **(C)** PLS-DA score plots (TG30 and rTG70); **(D,E)** heatmaps of the lipids content which at the top fifteen with VIP values of different comparison groups (D: TG30 and TG50; E: TG30 and rTG70).

The detailed EPA/DHA lipids composition is summarized in Figure 2, and the detailed statistical analysis of lipid molecules is displayed in Figure 3. It is well known that TGs are generally the main constituents of fish oil. The principal lipid of EPA/DHA in TG30, TG50, rTG50, and rTG70 was the TGs (99.70, 78.69, 98.20, and 88.44%, respectively), and accounted for the largest proportion of total lipid contents of EPA/DHA. These results showed that the EPA/DHA enrichment method did not alter its major. In addition, TG30 contained the highest amounts of TGs (99.70%). We propose that initial refinement processes which included degumming, to some extent, was able to remove some glyceride-type lipids such as monoglycerides (MGs) and diacylglycerol (DGs), as well as other polar lipids, for example phospholipids, which contributed to the high level of purity of TG lipids.

DGs were another dominant glyceride-type lipid, accounting for 0.23, 1.74, and 11.26% in TG30, rTG50, and rTG70, respectively, and were associated with the rising trend of EPA/DHA purity. DGs are predominant intermediates in the enzymatic synthesis of many lipids comprising of TGs. Dyerberg et al. (28) also discussed the influence of DGs content on EPA/DHA bioavailability. They proposed that, as DGs are composed of two fatty acids, reducing the stereospecific blockade could improve the hydrolysis efficiency of the chemical bonds, thus, facilitating the degradation of lipids and could potentially promote the formation of micelles.

The EPA/DHA contents as phosphatidylglycerols (PGs) and phosphatidylethanolamines (PEs) in TG50 were 12.58 and 4.99%, respectively, performed significant difference compared with TG30, rTG50, and rTG70 (*p*<0.05). PGs and PEs were regarded as the major glycerophospholipid components in TG50. These results were related to the EPA/DHA enrichment methods. During silica gel column elution, concomitant with the increase in solvent polarity and times, polar lipids also accumulated, in the meanwhile. Previous study (29) pointed that PGs and PEs could affect the development of cytomembranes, and also influence lipid metabolism by regulating the expression of associated proteins.

Both the phosphatidylinositol phosphate (PIP) and phosphatidic acid (PA) contents were at low levels in all samples. Moreover, contrary to what was observed with TGs and DGs, the MG contents were at reduced levels. Fatty acids (FAs) content in TG50 was 0.19%, higher than TG30, rTG50, and rTG70. Previous study (30) have found that the bioavailability of EPA/DHA as FAs was lower than as TGs.

Additionally, for better understanding the effect of different enrichment method on the EPA/DHA lipid molecules composition, detailed statistical analysis was displayed in Figure 3. Principal component analysis (PCA) results (Figure 3A) showed that EPA/DHA lipid molecules among the four TG type fish oils performed a quite clear separation, indicated that there were significant differences. Partial least squares discrimination analysis (PLS-DA) of Figures 3B, C also showed significant separation. The top fifteen differential lipid molecules of TG30 and TG50 group as well as TG30 and rTG70 group were exhibited in Figures 3D, E. As for TG30 and TG50 group, except for TGs, PGs and PEs were also differential lipid molecules. In addition, the top fifteen differential lipid molecules in TG30 and rTG70 group were all TGs.

### Cell viability and monolayer integrity assay

**FIGURE 4:**
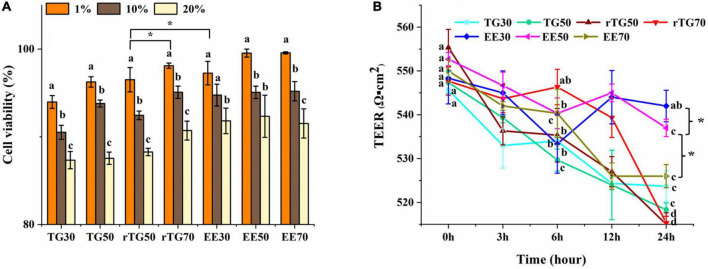
Evaluation of the cell viability and the Caco-2 monolayer cell membrane model. **(A)** Cell viability when incubated with mixed micelles for 24 h at different concentrations (1, 10 and 20%, v/v). **(B)** Changes in TEER values of Caco-2 cell monolayer after adding mixed micelles (20% concentration, v/v) and incubating for 0, 3, 6, 12 and 24 h. Data represents mean ± SD (*n* = 3). Different letters and * represent significant differences (*p*<0.05).

The principle of CCK-8 is that in viable cells, water-soluble tetrazolium salts (WST) will be restored into formazan by mitochondrial dehydrogenases (15). The amount of formazan formed is proportional to the number of viable cells. During the GIT simulation, previous studies have reported that pancreatic enzymes and bile salts can be cytotoxicity (15). And in our study, results showed that when the mixed micelles concentrations were lower than 20% (v/v) in TGs and EEs groups, the cell survival rates could maintain over 80% (after treating 24 h by mixed micelles) (Figure 4A) (15, 26). Therefore 20% (v/v) of mixed micelles were selected to treat cells in subsequent analysis.

As Figure 4A displayed that, rTG70 showed the highest survival rate at 20% (v/v) concentration of mixed micelles among TGs groups, and was significantly higher than rTG50 (*p*<0.05), we propose that high contents of PUFAs may increase fluidity, reduce deposition of pancreatic enzymes and bile salts during the GIT simulation.

TEER value is a key factor for determining the cell monolayers integrity. After cultivating for 21-days, the TEER value reached 560 Ω^⋅^cm^2^ which indicating that the Caco-2 cell monolayer had good integrity (24, 31). After being treated with mixed micelles (20% concentration, v/v) for 3, 6, 12, and 24 h (Figure 4B), the TEER values for each well were still about 510 Ω^⋅^cm^2^, which showed that Caco-2 cells still possessed monolayer model that meets the requirements (30). Moreover, after being treated for 24 h, the TEER values for EE30 and EE50 (about 520-540 Ω^⋅^cm^2^) were significantly higher (*p*<0.05) than EE70 (about 526 Ω^⋅^cm^2^) and other TG groups (about 510-520 Ω^⋅^cm^2^), which could be related to the lower content of fish oil in EE30 and EE50 mixed micelles (32).

### Concentration of EPA/DHA on the apical side

**FIGURE 5:**
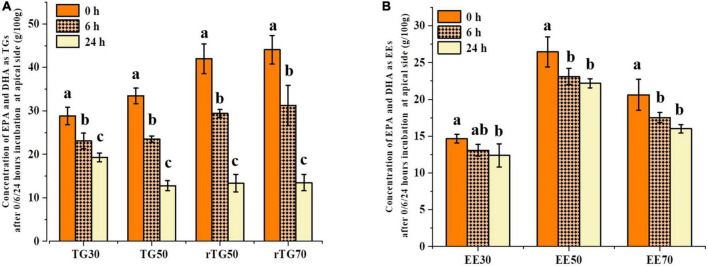
Concentrations of EPA/DHA as **(A)** TGs and **(B)** EEs on the apical side after 0, 6, and 24 h transport. Data represents mean ± SD (*n* = 3). Different letters (a, b, and c) indicate statistically significant differences among different groups by ANOVA (*p* < 0.05).

Changes in EPA/DHA concentrations at the apical side after 6 h and 24 h could reflect the cell transport capacity (Figure 5) (33). It is worth to note that the initial (0 h) concentrations of EPA/DHA on the apical side were not the same and this could be attributed to the continuously processes of GIT and Caco-2 cell transportation. As such, during this experiment design, the mixed micelles which obtained via the normal order of digestion should be regard as the starting point of transport (34, 35). After incubating for 24 h, EPA/DHA contents as TGs and EEs were significantly different compared with 0 h, implying that cell transport had occurred (Figures 5A, B). As expected, rTG70 expressed the highest cell transport capacity for EPA/DHA. The content of EPA/DHA on the apical side reduced from 44.06 g/100 g (0 h) to 13.48 g/100 g (24 h), a decrease of 69.41%, while rTG50 and TG50 decreased by 68.19% and 61.82%, respectively. As for EE30 and EE70, after transportation for 24 h, the concentrations of EPA/DHA on the apical side decreased by 15.51% and 22.41%, respectively, suggesting that both the purity and formulations of EPA/DHA could affect cell transport across monolayers.

### Cellular uptake

**FIGURE 6:**
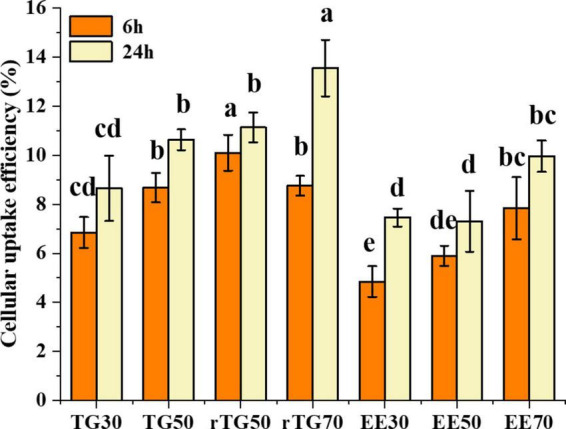
Cellular uptake of EPA/DHA as TGs and EEs after incubation for 6 h and 24 h. Data represent mean ± SD (*n* = 3). Different letters (a, b, c, and d) indicated statistically significant differences among different groups by ANOVA (*p* < 0.05).

Cellular uptake does not adhere to a similar concept as that of cell transport as well as bioavailability. During the cell transport process, a large proportion of EPA/DHA from the apical side was transported to the basal side, but a small portion was still stored inside the cell, indicating the capacity for cellular uptake, which could provide the foundation for bioavailability studies. As shown in Figure 6, cellular uptake of EPA/DHA as TGs and EEs was time-dependent at different purity levels. After 6 h incubation, rTG70 (8.76%) and EE70 (7.84%) were significantly higher than TG30 (6.85%) and EE30 (4.85%). In addition, rTG50 demonstrated an exceptional capacity for cellular uptake compared to rTG70, implying that the higher content of PUFAs in rTG70 did not increase their capacity for solubilization of lipophobic ingredients over a short time. However, after 24 h incubation, the cellular uptake of rTG70 and EE70 reached 13.55 and 9.96%, which were 1.57 and 1.34 times higher than TG30 (8.65%) and EE30 (7.46%), respectively. Furthermore, there was no significant difference between TG50 and rTG50.

### Bioavailability of EPA/DHA

**FIGURE 7:**
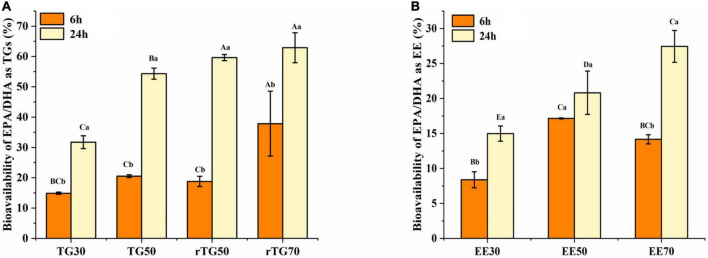
The bioavailability of EPA/DHA as **(A)** TGs and **(B)** EEs with different purities. Data represents mean ± SD (*n* = 3). Different letters (a, b) indicate statistically significant differences among 6 h and 24 h transport time (*p* < 0.05). Different capital (A, B, C…) represent significant differences among the 7 fish oils at the same transport time.

In the above series of experiments, the impact of purity and formulations on the *in vitro* bioavailability of EPA/DHA were determined (Figures 7A, B). Results showed that, after 6 h incubation, EPA/DHA bioavailability in both TGs and EEs groups did not display obvious regularity, this might be related to the short incubation time. As for the formulations, only rTG70 significantly higher than EE70 (*p* < 0.05).

After 24 h incubation, the bioavailability of EPA/DHA increased significantly (*p* <0.05) in parallel with the increase in purity for both TGs and EEs groups. EPA/DHA bioavailability for rTG70 (62.89%) and EE70 (27.44%) was about two folds higher than TG30 (31.72%) and EE30 (14.98%). Moreover, bioavailability for EE50 (20.81%) was significantly lower (*p* <0.05) than TG50 (54.33%) and rTG50 (59.64%), which indicated that at the same purity level, bioavailability of EPA/DHA as EEs was lower than TGs. In addition, there was no significant difference (*p* <0.05) between rTG70 (62.89%) and rTG50 (59.64%). This indicated that above a certain level, EPA/DHA bioavailability might not improve even with increasing purity.

For TG50, after transporting for 6 h, bioavailability was 20.53%, which was not significantly different compared to TG30 (14.90%) and rTG50 (18.79%). Interestingly, the bioavailability of TG50 was 54.33% after 24 h of incubation, which was significantly better compared to TG30 (31.72%), but not when compared with rTG50 (59.64%). TG50, with 12.58% of PGs and 4.99% of PEs, could theoretically improve EPA/DHA bioavailability, because PGs and PEs play a role in the construction of biological membranes. However, for this case, we predict that during the GIT process, no appropriate enzyme specifically hydrolyzed PGs and PEs, thus delaying the release of EPA/DHA as PGs and PEs. The GIT process may have also required other conditions such as time and presence of hydrolases to integrate PGs and PEs into the cell monolayers.

The ratios of EPA and DHA in the seven fish oil samples are presented in Supplementary Table 1. In this study, we did not place much emphasis on maintaining a consistent EPA and DHA ratio in the fish oils as this study was *in vitro* test. Moreover, ensuring the consistency of the EPA/DHA ratio would require mixing various fish oils prepared using different methods, which could betray the purpose of our study. Additionally, as absorption of fatty acids by the small intestine takes only a few hours, we selected 24 h to complete the process so that we could easily observe differences between samples. The extended time period also compensated for the inability of Caco-2 cells to perform intestinal peristalsis (24, 36).

In this study, the purity of EPA/DHA obtained through silica gel column purification was about 50% and also contained a small part of phospholipids. However, this level of purity was lower than rTG70 which obtained through re-esterification. Therefore, future studies are required to optimize the process of silica gel column purification to further improve EPA/DHA purity.

## Conclusion

Results from this study revealed that different EPA/DHA enrichment methods affect its lipid subclasses and lipid molecules composition differently. Enzymatic-based methods increased the content of EPA/DHA as DGs, while silica gel column purification improved the content of EPA/DHA as PGs and PEs. However, both methods did not change the major lipid of EPA/DHA as TGs. Data from the Caco-2 monolayer cell membrane transport model demonstrated that increasing EPA/DHA purity was a reliable way to enhance its bioavailability. Moreover, after 24 incubation, at the same level of purity, bioavailability of EPA/DHA as EEs was lower than EPA/DHA as TGs. Taken together, this study could promote the use of enzymatic-based preparation for production of high-quality fish oil and also demonstrated that the silica gel column method is a promising approach for EPA/DHA enrichment. This new approach also provides a basis for further investigations on the biological activity of fish oil with enhanced purity and bioavailability of EPA/DHA for application in the health care industry.

## Data availability statement

The data presented in this study are deposited in the MetaboLights repository, accession number: MTBLS6924.

## Author contributions

RS and WL: data curation and writing–original draft. SD: visualization and investigation. YZ: supervision. NT: supervision and writing–review and editing. All authors contributed to the article and approved the submitted version.
