# Supplementary material for: Assessment of lipid composition and eicosapentaenoic acid/docosahexaenoic acid bioavailability in fish oil obtained through different enrichment methods
Source: Front Nutr. 2023 Mar 14;10:1136490. doi: 10.3389/fnut.2023.1136490 (PMC10043196; doi:10.3389/fnut.2023.1136490)
Supplement: Supplementary file 1 [file Table_1.DOCX]

Supplementary Material

**Table S1** Ratio of EPA and DHA in each fish oil sample

| Samples | Ratio of EPA and DHA | EPA/TFA (%) | DHA/TFA (%) |
| --- | --- | --- | --- |
| TG30 | 1.56 ± 0.02 | 20.79 ± 0.44 | 13.30 ± 0.48 |
| TG50 | 2.18 ± 0.04 | 25.77 ± 0.14 | 11.81 ± 0.24 |
| rTG50 | 1.69 ± 0.05 | 33.55 ± 0.84 | 19.81 ± 0.46 |
| rTG70 | 1.82 ± 0.03 | 47.80 ± 0.64 | 26.28 ± 0.11 |
| EE30 | 1.63 ± 0.25 | 21.18 ± 1.49 | 13.13 ± 1.10 |
| EE50 | 0.54 ± 0.00 | 19.04 ± 0.07 | 35.06 ± 0.18 |
| EE70 | 0.26 ± 0.01 | 15.01 ± 0.39 | 58.86 ± 1.42 |

Values are mean ± SD, n = 3.
